# Harnessing Microalgae as Sustainable Cell Factories for Polyamine-Based Nanosilica for Biomedical Applications

**DOI:** 10.3390/molecules30081666

**Published:** 2025-04-08

**Authors:** Sik Yoon, Boon Huat Bay, Ken Matsumoto

**Affiliations:** 1 Department of Anatomy, School of Medicine, Pusan National University, Yangsan 50612, Republic of Korea; 2 Research Institute for Convergence of Biomedical Science and Technology, Pusan National University Yangsan Hospital, Yangsan 50612, Republic of Korea; 3 Department of Anatomy, Yong Loo Lin School of Medicine, National University of Singapore, Singapore 117594, Singapore; antbaybh@nus.edu.sg; 4 Chemical Genomics Research Group, RIKEN Center for Sustainable Resource Science, Saitama 351-0198, Japan; 5 Drug Discovery Seed Compounds Exploratory Unit, RIKEN Center for Sustainable Resource Science, Saitama 351-0198, Japan

**Keywords:** marine biotechnology, polyamines, diatoms, biofabrication, biosilica, nanocarriers, bioimaging, bone tissue engineering

## Abstract

Microalgae are microscopic unicellular organisms that inhabit marine, freshwater, and moist terrestrial ecosystems. The vast number and diversity of microalgal species provide a significant reservoir of biologically active compounds, highly promising for biomedical applications. Diatoms are unicellular eukaryotic algae belonging to the class Bacillariophyceae. They possess intricately structured silica-based cell walls, which contain long-chain polyamines that play important roles in the formation of silica. Long-chain polyamines are uncommon polyamines found only in organisms that produce biosilica. Diatomite, which is a marine sediment of the remains of the silica skeleton of diatoms, could be an abundant source of biogenic silica that can easily be converted to silica particles. This concise review focuses on the biofabrication of polyamine-based nanosilica from diatoms and highlights the possibility of utilizing diatom biosilica as a nanocarrier for drug and siRNA delivery, bioimaging, and bone tissue engineering. The challenges that may affect diatom production, including environmental stresses and climate change, are discussed together with the prospect of increasing diatom-based biosilica production with the desired nanostructures via genetic manipulation.

## 1. Introduction

Marine biotechnology holds significant potential for addressing major societal challenges by harnessing the vast biological diversity of marine ecosystems [1]. Microalgae are valuable alternatives to higher plants for various applications, owing to their rapid growth rates, versatile uses, and extensive biodiversity [2]. The estimated number of microalgal species ranges from 200,000 to 800,000, although only approximately 35,000 species have been documented [3].

Microalgae are typically unicellular photosynthetic eukaryotic organisms with a size range from 2 to 50 μm [4]. Although prokaryotic cyanobacteria (blue-green algae) are not strictly classified as microalgae, they share characteristics such as their photosynthetic ability, physiology, and ecological roles [5,6]. This often leads to their inclusion in the broader category of microalgae.

Microalgae inhabit marine, freshwater, and moist terrestrial ecosystems and represent a predominant component of marine and freshwater phytoplankton [7,8]. They play a crucial role in the productivity, diversity, and function of aquatic ecosystems, contributing to at least 32% of the earth’s photosynthesis, with up to 25% of global productivity, and 50% of all aquatic productivity, making them a significant component of the world’s biomass [9,10,11,12]. Unlike higher plants, microalgae lack true leaves, stems, or roots; however, they possess chlorophyll pigments essential for capturing solar energy and CO_2_ fixation [13].

Microalgae provide a vast reservoir of compounds with applications in biotechnology and industry, including food, health, energy, biomaterials, cosmetics, and environmental management [11,14,15]. Common microalgae used in biomedical applications include eukaryotic microalgae such as Chlorophyta like *Chlorella* and *Chlamydomonas reinhardtii*, as well as prokaryotic Cyanobacteria like *Limnospira platensis* (formerly *Spirulina platensis*) [16,17]. Microalgae can be efficiently cultivated in large-scale photobioreactors to produce significant amounts of biomass with rapid generation times, making them a renewable and largely untapped resource for developing drugs and other valuable bioactive agents [15,18].

In comparison with higher plants, microalgae are known to exhibit higher photosynthetic efficiency and resilience to environmental changes, producing a wide array of valuable bioactive compounds beneficial to human health, such as proteins, lipids, carbohydrates, pigments, vitamins, and alkaloids in significant quantities [19,20,21,22,23,24,25]. They are often referred to as “sustainable biofactories” due to their ability to mitigate CO_2_ and produce high-value compounds [26]. Currently, more than 15,000 new compounds derived from microalgal biomass have been chemically identified [27].

This concise review focuses on polyamine-based nanosilica, which is present in the cell walls of eukaryotic microalgae diatoms. Moreover, it highlights the potential biomedical applications of diatom-derived nanosilica that include drug delivery systems, bioimaging such as magnetic resonance imaging (MRI) and positron emission tomography (PET), and bone tissue engineering. Furthermore, the possible challenges and future perspectives of the diatom field, including the production and utilization of diatom biosilica are delineated.

## 2. Polyamines

Polyamines are aliphatic polycations containing two or more amine groups. Common polyamines such as putrescine, spermidine, and spermine are highly abundant in mammalian cells. They are essential for various cellular processes and confer various health benefits to multicellular organisms; thus, abnormalities in intracellular and systemic polyamine levels are associated with dysregulated cellular metabolism and pathology [28,29,30,31,32]. In this section, we summarize the intracellular functions of polyamines and their roles in human health, as well as the distinct classes of polyamines found in organisms that possess silica-based cell walls.

### 2.1. Polyamine Functions

Polyamines regulate various cellular functions, thus playing pivotal roles at the organismal level. Polyamines are essential for cellular proliferation and are also involved in translational regulation, autophagy, mitophagy (selective degradation of mitochondria via autophagy), regulation of the mitochondrial function, including beta-oxidation, and scavenging of reactive oxygen species, and interaction with ion channels [29,30,33,34].

Owing to their positive charges at physiological pH, polyamines in cells or living organisms are mostly bound to negatively charged macromolecules such as DNA, RNA, phospholipids, and ATP [35]. Polyamines are also implicated in the translational control of a set of mRNAs by binding to them and ribosomes, and modifying the secondary structures of mRNAs [33,36].

Spermidine has been reported to induce autophagy in yeast, flies, worms, and cultured human immune cells [37] via several potential molecular mechanisms [38]. Spermidine inhibits acetyltransferase EP300, which acetylates and inactivates several autophagy-related genes (ATGs). In addition, the activation of polyamine catabolism reduces protein acetylation by depleting the acetyl-CoA pools. eIF5A, which is activated via spermidine-mediated hypusination (see Section 2.4), facilitates the translation of ATG3 and the pro-autophagic transcription factor EB (TFEB). Since autophagy removes misfolded or aggregated proteins and damaged organelles, spermidine is involved in cellular homeostasis and proteostasis [30].

In tumor-bearing aged mice, spermidine supplementation improves the responsiveness to programmed death-ligand 1 (PD-L1) monoclonal antibodies [39]. Moreover, spermidine supplementation enhances mitochondrial functions, with the mitochondrial activity of T cells being associated with antitumor activity. By searching for proteins that bind to spermidine, the mitochondrial trifunctional protein responsible for beta-oxidation of fatty acids in mitochondria was identified as a spermidine-binding protein. Spermidine activates fatty acid oxidation in vitro and in vivo [34,39]. Furthermore, spermidine treatment improves mitochondrial fatty acid oxidation in mouse models of non-alcoholic steatohepatitis (NASH) [40].

The functions of polyamines in living organisms, including anticancer, anti-aging, and immune regulation, have been extensively studied. Polyamine levels are generally high in cancer cells with high proliferative abilities [41,42]. Therefore, reducing the levels of polyamines in cancer cells is a promising strategy for cancer treatment. Treatment with the ornithine decarboxylase (ODC) inhibitor, difluoromethylornithine (DFMO; eflornithine), reduces the intracellular levels of putrescine and spermidine. However, the intake of polyamine does not induce cancer in healthy individuals [43], whereas supplementing food with spermidine decreases the incidence of cancer [44,45].

In mice, the polyamine content declines with age [30,39,46]. Aging induces aging-associated pathologies such as cardiovascular diseases, metabolic diseases, cancers, and neurodegenerative disorders, including cognitive impairment. When supplemented to aging animals, spermidine acted as a caloric restriction mimetic, targeting aging-associated pathologies [30]. The main anti-aging action of spermidine is via the induction of autophagy; it induces mitophagy in cardiomyocytes and various cultured cell lines, which contributes to the maintenance of mitochondrial functionality [30]. The role of polyamines in autophagy induction and anti-inflammation contributes to the prevention of cardiovascular diseases [32,47]. Furthermore, polyamines improve cognitive functions in animal models [48,49]. Consistent with these results, polyamines have life-extending effects when supplemented to aged animals [37,50,51].

### 2.2. Synthesis, Uptake, and Export of Polyamines

The intracellular concentrations of polyamines are regulated via intracellular biosynthesis, uptake from the environment, and export of polyamine metabolites.

**Figure 1:**
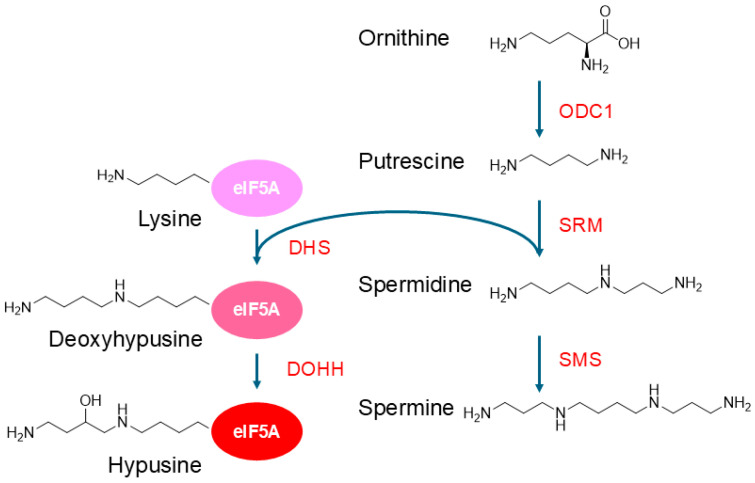
Pathways of polyamine synthesis and hypusination of eIF5A. Hypusination is essential for the activity of eIF5A. ODC1, ornithine decarboxylase 1; SRM, spermidine synthase; SMS, spermine synthase; DHS, deoxyhypusine synthase; DOHH, deoxyhypusine hydroxylase.

Polyamines derive from amino acids and are synthesized in the cytoplasm via the polyamine synthetic pathway, which is closely linked to the urea cycle (Figure 1). Putrescine is synthesized from ornithine by ODC, which is the rate-limiting enzyme in this pathway. Other diamines, cadaverine and agmatine, are synthesized via the decarboxylation of lysine and arginine, respectively. Spermidine and spermine are synthesized from putrescine through consecutive aminopropyl transfer reactions by spermidine and spermine synthases, respectively, with the aminopropyl groups being provided by decarboxylated S-adenosylmethionine (dcSAM or deAdoMet) [28,29].

The uptake of polyamine from outside the cells is mediated by various transporters, including solute carriers and P-type ATPases [52,53]. SLC7A5, SLC22A2, and SLC18B1 are solute carrier family polyamine transporters localized in the plasma membrane or vesicles [54,55,56]. Polyamines are also imported via endocytosis. They are transported into the cytoplasm by the P-type ATPases ATP13A3 and ATP13A2, which localize to early endosomes and late endosomes/lysosomes, respectively [53].

In the catabolic pathways of polyamines, spermine is converted to spermidine by spermine oxidase, with hydrogen peroxide and acrolein being produced as toxic by-products [31]. Spermidine and spermine are acetylated by spermidine/spermine-*N*(1)-acetyltransferase (SSAT). *N*1-acetylspermidine and *N*1-acetylspermine are partially converted to putrescine and spermidine, respectively, by polyamine oxidase; however, they are also excreted from cells. The solute carrier family transporter SLC3A2 mediates the uptake of putrescine in inside-out membrane vesicles prepared from putrescine-tolerant CHO cells, suggesting that this transporter also mediates putrescine export from cells [57,58].

**Figure 2:**
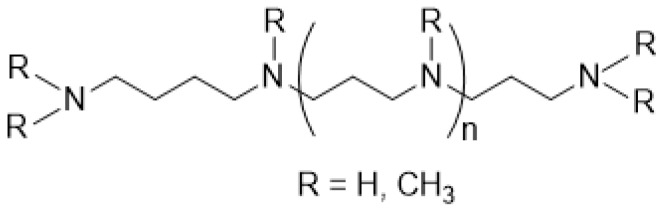
Representative structure of LCPAs.

Organisms that form biosilica, such as diatoms, marine sponges, and spore-forming bacteria, synthesize long-chain polyamines (LCPAs) (Figure 2) [59,60,61,62].

In many cases, the long chains of LCPAs are composed of multiple *N*-methylated aminopropyl groups. However, LCPAs containing extended chains with aminobutyl groups have been also reported [63]. Diatom genomes carry gene fusions of *S*-adenosylmethionine decarboxylase, which produces dcSAM, and an aminopropyltransferase that potentially synthesizes LCPA. Furthermore, in some cases, they are fused to a histone *N*-methyltransferase domain that *N*-methylate LCPA [64,65]. LCPAs extracted from diatom biosilica demonstrated in vitro silica precipitation activities [59].

### 2.3. Polyamine Intake

Animals take up polyamines as nutrients from two sources: food and gut microbiota. Polyamines are transferred to the blood through the intestine lumen [66] and exhibit anti-aging activities; therefore, it is important to increase the polyamine intake from food or microbiota for a healthy long life [47]. Spermidine and spermine are common in both plant- and animal-derived foods, whereas putrescine is abundant in fermented products. Polyamines in plant-derived foods occur either as free molecules or conjugated with phenolic compounds, such as coumaric and caffeic acids [32,67]. Spermidine supplements are commercially available, primarily derived from wheat germ. The upregulation of polyamine production in the gut microbiota also aids the intake of polyamines [51]. This can be accomplished by single or combinations of polyamine-producing bacteria [34,66]. Thus, polyamines from food and those produced by bacteria in the intestine contribute not only to nutrient intake but also to host–bacteria communication and host immunity.

### 2.4. Post-Translational Modification of Proteins with Polyamines

An important role of spermidine is to supply the aminobutyl moiety for the hypusination of the translation factor eIF5A [68,69,70]. Hypusination is a post-translational modification that only occurs to a specific lysine residue (Lys50 in humans) of the eIF5A protein (see also Figure 1). Hypusination is mediated by two enzymatic reactions: transfer of the aminobutyl moiety of spermidine to the lysine in eIF5A and subsequent hydroxylation [68]. Although eIF5A was originally identified as a translation initiation factor, it is now known to play a role in translation elongation and termination by binding to the ribosomes between the P-site and E-site, where the hypusinated eIF5A interacts with the P-site tRNA and facilitates the peptidyl transfer reaction of difficult-to-translate sequences [68,69]. The hypusinated eIF5A plays a role in promoting mitochondrial respiration and function; however, excess eIF5A, whether hypusinated or not, deregulates the mitochondrial function [71,72,73].

In diatom cell walls, biosilica contains peptides called silaffins that are able to precipitate silica in vitro [74,75]. Silaffins are modified with LCPAs at specific lysine residues in addition to *N*-trimethylation at other lysine residues and phosphorylation at serine residues [74,76]. These modifications facilitate the precipitation of silica [76].

Aculeines, which are polypeptides with LCPA-modified N-terminal residues, were isolated from the marine sponge *Axinyssa aculeata*. Aculeines induce convulsions in mice upon intracerebroventricular injection and disrupt neuronal membrane integrity [77].

## 3. Biofabrication of Polyamine-Based Nanosilica from Microalgae

### 3.1. Formation of Biosilica in the Cell Walls of Microalgae

**Figure 3:**
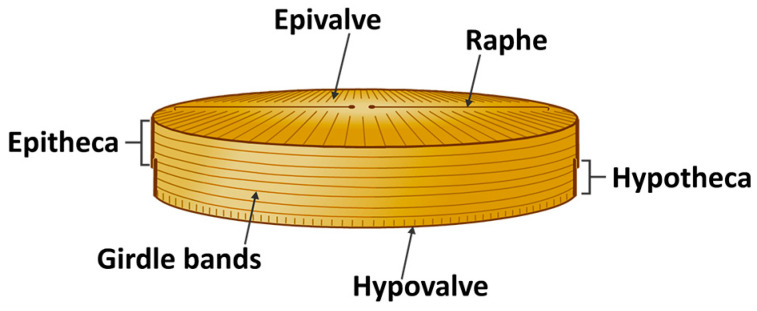
Morphology of diatoms.

Diatoms are unicellular eukaryotic algae belonging to the class Bacillariophyceae, possessing silica-based cell walls with complex structures [78]. At least 100,000 diatom species exist [79], making them the most copious and varied siliceous marine microorganisms [80]. The word “diatom” is a Greek word that means “cut into two”, whereby in this case the two halves are the epitheca, comprising the epivalve and its copulae (thin bands of silica), and the hypotheca, containing the hypovalve and its copulae, with the appearance compared to that observed in a pillbox or Petri dish (Figure 3). In several species, the valves have multiple layers with each layer typified by distinct porous patterns [81,82]. The silicified cell wall, known as the frustule, differentiates centric diatoms with circular or polygonal valves from pennate diatoms with elongated frustules [83]. An opening or slit in the silica cell wall is called raphe.

For more details on the biogenesis of the diatom cell wall, please refer to Kröger and Poulsen [84]. Briefly, as cells undergo cytokinesis during division, new biosilica is formed inside the valve of the silica deposition vesicles (SDVs), which enlarge with increasing silica deposition. Finally, each SDV containing a fully developed valve is deposited in a cleavage furrow (groove on the plasma membrane) during cell division on the surface of each protoplast by exocytosis. As the sibling cells separate and enter the interphase, the expansion of the protoplast necessitates the synthesis of new biosilica.

**Figure 4:**
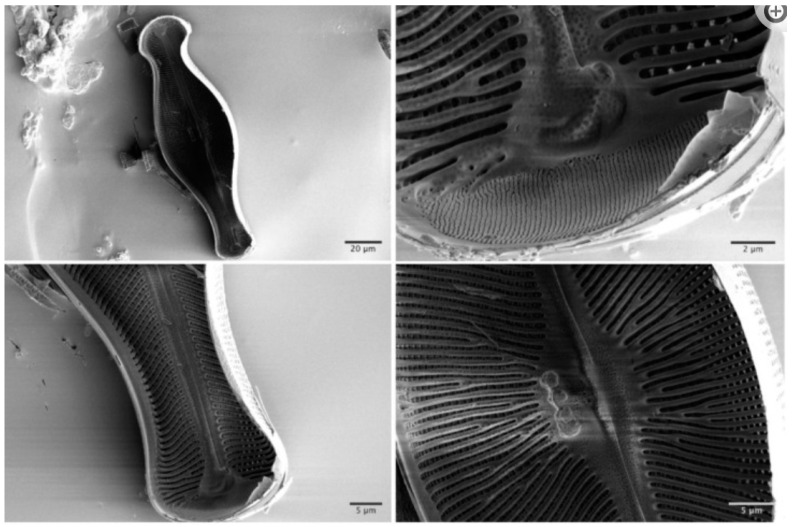
Scanning electron microscopy images of the inner structures of *Didymosphenia geminata* after removal of the epivalve part from the hypovalve part. Reprinted from Topal et al. [86].

An example of the intricately sculptured silica cell wall, which has been called a hallmark of diatoms [85], is shown in Figure 4 (reproduced from Topal et al. [86]).

**Figure 5:**
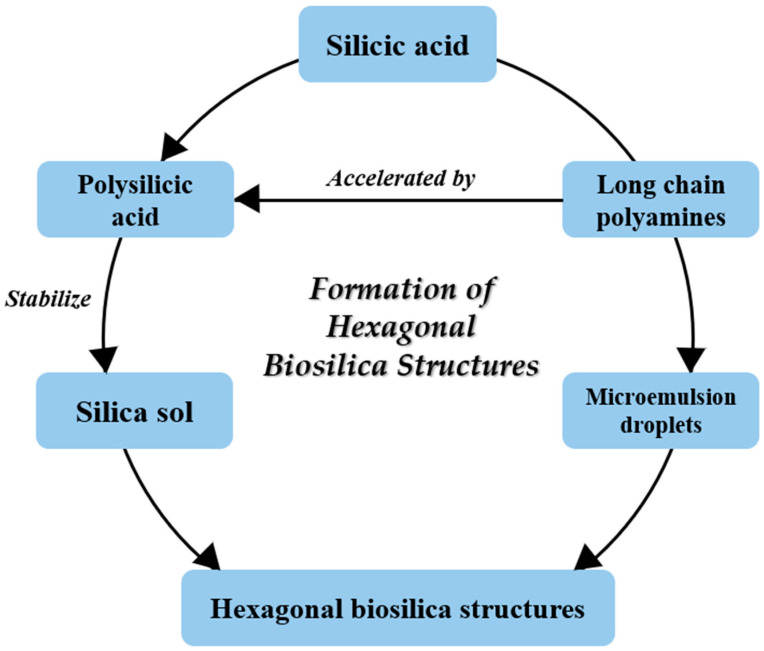
Formation of hexagonal biosilica structures in the cell walls of diatoms (according to Sumper [88]; Sumper and Lehmann [78]).

Biosilica from the diatom cell wall contains LCPAs that are attached to putrescine, spermidine, or silaffins [59,76,84]. LCPAs play important roles in the formation and shaping of silica [75,78,87]. A schematic of the formation of hexagonal silica structures in the diatom cell walls according to Sumper [88] and Sumper and Lehmann [78] is shown in Figure 5.

The rate of silica formation is possibly regulated by the degree of methylation of species-specific polyamines [78]. Once intracellular maturation occurs in SDV, the synthesized silica elements are exocytosed to cover the cell surface [89,90,91]. The transport of the silica building blocks from the environment to SDVs is an important part of the process, with diatoms maintaining a constant silicon pool [92]. Recently, Mayzel et al. [85] used cryoelectron tomography to image silica formation and revealed that diatom silification may also occur extracellularly.

The silica cell walls of diatoms serve several functions [84] acting as (a) an efficient and protective armor against planktonic predators, such as parasites [93] (b) a proton buffer that aids in CO_2_ acquisition [94], and (c) a photonic crystal, which could aid in light harvesting for photosynthesis [95,96]. Silicanin 1 (Sin 1), a protein encoded by the *Sin1* gene, known to be conserved in all groups of diatoms [97], has been shown to be essential for the biosynthesis of mechanically robust diatom cell walls [98].

In addition to biosilica, diatom cells also contain organic materials, and their formation occurs through a biofabrication process, which requires the synthesis of calcium-based and iron-based biominerals [96].

### 3.2. Diatom-Based Biosilica Production with Tailored Nanostructures via Genetic Manipulation

Diatoms have been genetically engineered to fabricate intricately structured biosilica with tailored nanostructures, enhancing their suitability for various biomedical applications [99,100]. Genetic modifications often target silaffins and cingulins—key proteins involved in biosilica formation [101,102]. Altering genes encoding these proteins enable control over the morphology and porosity of diatom biosilica, facilitating the synthesis of nanostructures with improved drug-loading capacity and controlled release properties. For instance, overexpression of silaffins increases silica deposition, while gene knockouts reduce silicification but enhance cell growth and photosynthetic efficiency [103]. These modifications significantly impact the physicochemical and optical properties of diatom frustules, expanding their potential applications. Furthermore, cingulin-containing microrings exhibit protein-based nanopatterns resembling the girdle band silica structures, providing insights into the morphogenesis and functionality of diatom biosilica [104]. These studies highlight the potential of genetic manipulation to fine-tune diatom biosilica properties for biomedical applications, particularly in drug delivery systems (please see also Section 4).

Recent developments in CRISPR-Cas9-based genome editing have further expanded the capabilities of diatom-based biosilica engineering. This precise tool allows targeted modification of biosilica-related genes, enabling fine-tuning of structural and chemical properties while ensuring efficient and stable gene mutations [105]. Such advancements enhance the ability to customize diatom biosilica for specific applications in advanced material design. Therefore, genetic manipulation of diatoms represents a promising approach for producing biosilica with tailored nanostructures, paving the way for innovative applications across diverse scientific and technological fields.

Beyond modifying endogenous genes, researchers have inserted foreign peptides or biomolecules into diatom biosilica using genetic fusion strategies, allowing functionalization with specific targeting ligands or bioactive compounds. For instance, a molecular genetic approach was successfully employed to integrate foreign proteins with specific functionalities into biosilica structures, enabling enzyme immobilization and enhancing their stability for various applications [106].

## 4. Biomedical Applications of Polyamine-Based Nanosilica from Diatoms

Diatom-based silica is available from diatom cell cultures and diatomite (also known as diatomaceous earth mineral) [107]. Diatomite, a marine sediment of the remains of the silica diatom skeleton, is an inexpensive and plentiful source of biogenic silica that can be easily converted to silica particles with controlled size and chemistry [108]. Diatomite micro- and nanoparticles can be derived from raw diatomaceous earth in three main steps, namely, crushing with a milling instrument, purification with acid to remove impurities, and size-based separation via sedimentation and filtration processes [82]. The use of diatom silica frustules for nanotechnological applications was first proposed by Gordon and Drum [109]. Durability, high mass transport rates, and large surface areas with interesting optical properties make diatom silica a useful nanomaterial for chemical sensing and catalysis, particularly in combination with microfluidics or lab-on-a-chip technologies [110,111,112], in addition to potential biomedical applications.

### 4.1. Diatom Biosilica as a Nanocarrier for Drug and siRNA Delivery

Diatoms contain naturally occurring biosilica, which can be used for developing cost-effective drug delivery systems with superior biocompatibility, large surface areas, and facile surface modification [113]. Losic et al. reported the use of Coscinodiscophyceae (*Aulacoseira* sp.) as nanovectors; the surface of diatoms was modified with dopamine and iron oxide nanoparticles (DOPA/Fe_3_O_4_/diatom composite), followed by loading with indomethacin (a non-steroidal anti-inflammatory drug) [114]. A year later, Aw et al. demonstrated the possibility of loading two drugs, hydrophobic indomethacin and hydrophilic gentamicin, into diatomite microcapsules and demonstrated their sustained release over two weeks [115]. A silica nanoporous biomaterial from the diatom *Coscinodiscus concinnus* (Coscinodiscophyceae) used as a nanocarrier for streptomycin, revealed a biphasic release of the antibiotic; an initial burst and a sustained release [116]. Vasani et al. [117] used oligo(ethylene glycol) methacrylate copolymer-modified diatom biosilica microcapsules for the controlled release of levofloxacin by thermo-responsive drug release and demonstrated the efficacy of the antibiotic against the bacteria *Staphylococcus aureus* and *Pseudomonas aeruginosa*.

Todd et al. [118] loaded dye molecules as drug mimics onto iron oxide nanoparticle-tagged diatoms and demonstrated in vivo their attraction to tumors under magnetic guidance. Moreover, diatom silica particles have been shown to enhance the permeability of aminosalicylate mesalamine and prednisone in colonic cells in vitro [119]. Genetically engineered *Thalassiosira pseudonana* (Mediophyceae) has been used to produce nanoporous biosilica for targeted drug delivery of anticancer drugs, where the biosilica surface was modified to display an IgG-binding domain of protein G, facilitating the attachment of cell-targeting antibodies that selectively bind to and eliminate neuroblastoma and B-lymphoma cells [120]. Furthermore, the same researchers encapsulated biosilica with camptothecin and its more potent derivative SN38, which was loaded onto liposomes and cetyltrimethylammonium bromide, respectively. They observed the killing of the neuroblastoma and B-lymphoma cells in vitro and the selective targeted delivery of chemotherapy drugs in vivo, achieving a significant decrease in tumor growth in SH-SY5Y neuroblastoma xenografts.

Diatom silica nanoparticles conjugated with polyethylene glycol (PEG) coverage and cell-penetrating peptides have been shown to enhance intracellular uptake in MCF-7 and MDA-MB-231 breast cancer cells, with higher biocompatibility and reduced cytotoxicity [121]. The same study reported the increased solubility of sorafenib loaded onto diatomite nanoparticles (DNPs) and its enhanced drug release profiles in aqueous solutions.

C-phycocyanin from Limnospira platensis exhibited enhanced cytotoxicity after *photodynamic therapy* when conjugated to diatom biosilica, as a biosilica photosensitizer to tumor-associated macrophages [122]. Furthermore, mesoporous silica derived from diatoms and loaded with fucoidan augmented the killing of HCT116 colon cancer cells via chemo-photodynamic therapy [123].

Rea et al. [124] demonstrated that diatom biosilica nanovectors could deliver siRNA to human epidermoid cancer cells, targeting the glyceraldehyde 3-phosphate dehydrogenase mRNA and achieving effective silencing. Furthermore, DNPs conjugated with an idiotype-specific peptide (endowed with high affinity toward the B-cell receptor) and loaded with siRNA directed against Bcl2 mRNA, for specific targeting of aggressive murine A20 lymphoma cells, resulted in significant gene silencing [125].

Surface modification of diatoms for achieving desired surface-enriched functionalities has profound implications for potentiating diatomite biosilica-based drug delivery [113,126].

### 4.2. Diatom Biosilica for Bioimaging and Biosensing

The frustule structure of diatoms has special optical effects, such as photoluminescence [95], with its size and stability being ideal for biomedical imaging and diagnostic applications [127]. Diatoms are also very promising for developing highly stable metal-silica hybrid nanoparticles with luminescent properties for bioimaging [128]. Mesoporous-derived biosilica nanostructures can be used for diagnostic imaging inMRI andPET [129], with hybrid-based porous silica nanoparticles being highly promising for both drug delivery and bioimaging [130].

Additionally, diatom biosilica functionalized with a fusion protein consisting of a ribose-binding protein flanked by fluorescent proteins (CyPet and YPet) enabled fluorescence resonance energy transfer (FRET)-based signaling in response to ribose binding, demonstrating its potential in biosensing applications [131]. Furthermore, to optimize biosilica-targeted fusion proteins for biosensor development, *Thalassiosira pseudonana* was genetically modified to express enhanced green fluorescent protein (EGFP), showcasing the feasibility of in vivo functionalization of diatom biosilica frustules [132].

### 4.3. Diatom Biosilica for Bone Tissue Engineering

Bone fractures, with an estimated 178 million new cases globally in 2019 [133], are a serious health issue, posing an economic burden to society [134,135]. Delayed union or nonunion is a known complication of failed fracture healing. A solution to this problem is the application of bone tissue engineering, an integrated platform that uses stem cells held together by a three-dimensional framework (scaffold) that provides the initial mechanical strength and induces molecular signaling pathways that mediate differentiation of progenitor cells into an osteoblastic phenotype [136]. Silicon is essential for bone health [137], and its linkage with glycosaminoglycans is important for the formation of cross-links between proteoglycans and collagen, which determine the strength, structure, and mechanical properties of the bone [138].

In light of the above, Le et al. [108] have advocated the use of diatom particles as a silica supplement for improving the osteoinductive features of scaffolds for bone tissue engineering in promoting healing of bone defects. In fact, sodium alendronate incorporated into diatom biosilica has been demonstrated to activate osteoblast-like cells and bone marrow stem cells and inhibit osteoclast-like cells [139]. Amoda et al. [140] revealed that biomaterials from the diatom *Cyclostephanos* sp. (Mediophyceae) frustules were biocompatible with the pre-osteoblast MC3T3-E1 cells and associated with positive staining for mineral deposits after 21 days. Furthermore, the addition of diatom particles to silk fibroin (a biological polymer used as a scaffold material for bone tissue regeneration) has been shown to enhance the osteogenic properties of osteoblast-like cells [141]. A 3D fibrous scaffold was developed for bone tissue engineering, produced via co-electrospinning poly(hydroxybutyrate-co-hydroxyvalerate)/poly(ε-caprolactone) fibers and diatom shell-incorporated pullulan (a water-soluble polysaccharide) fibers. It showed high osteocompatibility with Saos-2 human osteosarcoma cells, accompanied by improved cellular spreading and distribution [142].

Diatom biosilica is also a promising bone filler additive in 3D-printed poly (DL-lactide-co-glycolide) (PDLGA) scaffolds used in bone tissue engineering [143]. Moreover, diatom-PDLGA scaffolds exhibited more desirable physiochemical properties for bone repair than bioglass, which is a glass-ceramic biomaterial containing silicon dioxide [144]. Another advantage of using diatom frustules as a source of biosilica over bioglass is that the former is naturally synthesized, as opposed to the latter, which is fabricated under harsh conditions [145].

Chitosan/diatomite composite membranes prepared via solvent casting enhanced the spreading and proliferation of osteoblastic Saos-2 cells, together with increased alkaline phosphatase activity. Therefore, they could potentially be used in developing scaffolds for effective bone tissue engineering [146]. Recently, a GCH-Di-S scaffold produced by crosslinking gelatin, chitosan, and hyaluronic acid, loaded with diatom and β-sitosterol using the freeze-drying method, was observed to expedite new bone formation in a rat tibia defect model, thereby demonstrating the osteoconductive and osteoinductive properties of the scaffold [147].

### 4.4. Other Potential Biomedical Uses of Diatom Biosilica

Diatom biosilica has been touted as an inexpensive, non-immunogenic, and effective hemostatic agent that has advantages over other commonly used agents [148,149]. Feng et al. [149] showed that chitosan-coated diatom can induce erythrocyte absorption and aggregation, together with activation of the intrinsic coagulation pathway, thus quickening the blood clotting process and arresting further hemorrhage, which in some cases can lead to dire consequences. In a similar vein, diatom biosilica has been observed to promote wound healing [126]. Fabrication of diatom biosilica with hydroxbutyl chitosan hydrogel and loaded with the antibiotic doxycycline, has been reported to enhance wound re-epithelization in in vivo models [150]. Furthermore, diatom biosilica is not only useful for the treatment of acute wounds but also in the case of chronic wounds, such as diabetic wounds, as biomineralized silica has the propensity to aid angiogenesis and collagen deposition during the wound healing process [151]. Liu et al. [152] have also shown that diatomtaceous earth (containing biosilica) conjugated with zinc oxide exhibits enhanced antimicrobial activity against the fungus *Aspergillus fumigatus* and the Gram-negative bacteria *Escherichia coli* and *Salmonella enterica*.

## 5. Challenges and Perspectives

Diatom-based biosilica is recognized for its biocompatibility and shares morphological and functional similarities with synthetic silica, including its ability to focus light. As a result, it is considered a viable alternative to synthetic silica due to its natural abundance and cost-effective production [153]. Diatoms can be cultivated not only in laboratory settings but also on an industrial scale, enabling large-scale biosilica production [154]. A key advantage of diatom-based biosilica is its highly porous and modifiable surface, which allows for easy chemical functionalization tailored to specific biomedical applications, such as drug delivery [155]. Silanization is the most commonly employed method for the functionalization, as diatom biosilica contains hydroxyl groups that facilitate surface modification for biomedical device fabrication [155].

Currently, nanosilica can be synthesized through chemical processes such as precipitation, sol-gel synthesis, and spray pyrolysis [156]. Non-porous silica nanoparticles (SiNPs) are typically produced via flame hydrolysis from silicon tetrachloride, while colloidal SiNPs can be synthesized using sol-gel techniques, and porous SiNPs are obtained through microemulsion methods [157]. However, microemulsion-based SiNP synthesis can be costly due to the need for extensive purification and recovery processes for large-scale production [158]. Given that synthetic SiNPs require advanced nanofabrication facilities, whereas biogenic silica offers a low-cost, scalable alternative, diatom-based silica presents a compelling competitor to synthetic nanosilica for future nanomedicine applications [155].

One concern regarding silica materials, particularly amorphous silica, is their potential toxicity. Amorphous nanosilica has been implicated in inflammation, cytotoxicity, and immunological alterations [159]. However, numerous studies have demonstrated that diatom-derived biosilica exhibits low or negligible toxicity, making it a promising material for biomedical applications [119,160,161,162]. Notably, biosilica has shown selective cytotoxicity in drug delivery applications. For example, curcumin-loaded biosilica exhibited cytotoxic effects against human renal adenocarcinoma cells while sparing HEK293 human embryonic kidney cells, suggesting that biosilica can be engineered to selectively target cancerous cells without harming healthy ones [127].

Understanding the biodistribution of biosilica is crucial for evaluating its safety and efficacy. Research on mesoporous silica nanoparticles, which share similarities with diatom biosilica, has shown that their distribution across organs—such as the liver, spleen, and lungs—varies based on particle size and surface properties [163]. Additionally, the route of administration plays a significant role in determining biodistribution patterns.

The immune response to diatom biosilica is another critical consideration in biomedical applications. Studies have shown that mice injected with biosilica exhibited no significant inflammatory response or tissue damage in major organs, including the brain, heart, lungs, liver and kidneys [120]. The interaction of biogenic silica with the immune system is influenced by factors such as particle size, surface chemistry, and functionalization, highlighting the importance of optimizing these parameters to modulate immune responses [164,165]. Therefore, precise control over the physicochemical properties and functionalization of biogenic silica nanoparticles can help minimize immunogenicity, enhancing their safety and efficacy in biomedical applications. However, detailed studies specifically addressing the immune response to diatom biosilica remain limited, underscoring the need for further research to fully characterize its immunological profile.

Natural silica, such as diatom-derived biosilica, has been found to be more biodegradable and biocompatible than synthetic silica [99,148]. Studies have demonstrated that biosilica can be broken down in the body and excreted through the kidneys [120,166]. In drug delivery applications, diatom biosilica offers not only biocompatibility but also mechanical strength and chemical stability, which are crucial for maintaining drug carrier integrity under physiological conditions [167].

Biosilica is widely recognized for its chemical inertness and thermal stability, contributing to its durability in various environments [168,169]. However, its long-term stability in biological systems is influenced by factors such as pH, enzymatic activity, and interactions with biological molecules [167,170,171]. These factors, along with the chemical composition and structural properties of diatom biosilica, play a crucial role in determining its persistence and function in biological environments [96].

A deeper understanding of these factors is essential for evaluating the durability, versatility, and functionality of biosilica, supporting its use across a wide range of industrial and biomedical applications. While biosilica is generally considered stable, its long-term persistence and potential accumulation in tissues warrant further investigation to ensure its safety in biomedical application. Moreover, while diatom-derived biogenic silica presents a promising platform for biomedical applications due to its biocompatibility, low toxicity, and unique structural properties, further research is needed to fully assess its safety. Comprehensive studies on its potential adverse effects—including immune response, detailed biodistribution, and long-term stability in vivo—are essential to optimize its design for specific therapeutic applications.

The pattern formation of species-specific silica-based cell walls in diatoms is an attractive research field, not only for biological morphogenesis, but also for biofabrication. Diatom biosilica formation occurs in SDVs, where liquid–liquid phase separation mediated by proteins and LCPAs plays an important role in pattern formation [88,172]. In vitro reconstitution experiments have shown that organic components, including LCPAs and soluble proteins isolated from the diatom *Stephanocyclus cryptica* (synonymous with *Cyclotella cryptica*) facilitate the synthesis of porous silica patterns with silicic acid onto insoluble matrices [173]. The pattern of the reconstituted silica was similar to that of biosilica isolated from the same species. Although LCPAs play a critical role in silica pattern formation, it remains unclear whether they are responsible for the formation of species-specific silica patterns. Proteomic analysis and genetic manipulation have shown that the Sin 1 family of proteins is critical for silica pattern formation [172]. To gain more insight into the silica pattern formation in diatoms, further identification of the proteins that localize to SDVs and function in the biosilica formation is required, together with functional analysis by gene disruption of them and of the enzymes that are likely responsible for the synthesis of LCPAs. Such analyses may lead to the mass production of biosilica with desired nanostructures [61].

Environmental stressors such as light and nutrient deficiency can affect the productivity of diatoms [174]. The marine environment is faced with the incessant problem of increasing pollution, especially metal pollution [175], which has impacted the production of diatoms. Climate change is known to affect all ecosystems, affecting the survival of species [176]. The marine environment is in danger due to increasing temperature and diminishing salinity [177], as well as ocean acidification due to increased anthropogenic emissions of CO_2_ from the burning of fossil fuels and deforestation [178]. Climate change also affects phytoplankton dynamics (including diatoms) impacting bloom timing and community composition [179].

Hence, to optimize costs and ensure the sustainability of diatom production, attention should be paid to breeding, culturing, and harvesting [180]. For instance, the use of light-emitting diode (LED)-based photobioreactors coupled with advanced synthetic biology approaches could potentially enhance the growth performance and increase the productivity of diatoms [174]. Furthermore, to attain higher economic benefits, the possibility of co-extracting other valuable products from diatoms, such as chrysolaminarin and eicosapentaenoic acid should be considered [180]. The interspecies variation in the silica content depends on various factors, including size and growth patterns, as well as environmental influences, such as light, temperature, nutrients, and salinity. Therefore, detailed investigations of these variables may yield valuable insights into diatom-based silica [181,182]. Research on downstream processes, such as the development of a more efficient extraction process of biosilica from diatoms, is an important area for future exploration. Climate change issues would require an integrated approach with solutions such as biodiversity conservation, adaptation interventions, and protection of ecosystems [183], where such mitigation strategies would also require concerted intergovernmental efforts.

## 6. Conclusions

Diatoms are unicellular microalgae which are present in abundance in marine environments. Being a vital part of the marine ecosystem, they are involved in several processes, such as oxygen production and serving as a part of the marine food chain, which are essential for the survival of aquatic organisms. As silicifying organisms, diatoms possess ornate cell walls or frustules composed of polyamine-based biosilica. Interestingly, polyamines are essential in a myriad of cell functions, and LCPAs in particular, enable modification of silaffin proteins, thereby facilitating silica precipitation, and also in the shaping of the unique silicified frustules. Diatoms can be harvested in the living state or as fossil sediments, and therefore are environmentally sustainable green factories for producing biosilica for a multitude of commercial and industrial uses, with potential applications in the biomedical field such as diagnostics, drug delivery and bone tissue engineering. Indeed, diatom biosilica shows great promise as cost-effective theranostics.

## Data Availability

Not applicable.
